# The West African Health Organization’s experience in improving the health research environment in the ECOWAS region

**DOI:** 10.1186/s12961-016-0102-7

**Published:** 2016-04-20

**Authors:** Jude Aidam, Issiaka Sombié

**Affiliations:** West African Health Organisation, 01 BP 153, Bobo-Dioulasso, 01 Burkina Faso

**Keywords:** West Africa, Research environment, Research development experience, WAHO

## Abstract

**Background:**

The West African Health Organization (WAHO) implemented a research development program in West Africa during 2009–2013 using the Knowledge for Better Health Research Capacity Development Framework, developed by Pang et al. (Bull World Health Organ 81(11):815–820, 2003), on strategies used to improve the research environment. The framework has the following components: stewardship, financing, sustainable resourcing and research utilization. This paper describes how WAHO implemented this research development program in the West African region to help improve the research environment and lessons learnt.

**Methods:**

This is a retrospective review of the regional research development program using a triangulation of activity reports, an independent evaluation and the authors’ experiences with stakeholders. This program was designed to address gaps along the components of the framework and to improve partnership. The activities, results and challenges are summarised for each component of the framework. The independent evaluation was conducted using over 180 semi-structured interviews of key stakeholders in the West African region and activity reports. WAHO and major stakeholders validated these findings during a regional meeting.

**Results:**

All 15 ECOWAS countries benefited from this regional research development program. WAHO provided technical and financial support to eight countries to develop their policies, priorities and plans for research development to improve their research governance. WAHO, along with other technical and financial partners, organised many capacity-strengthening trainings in health systems research methodology, resource mobilization, ethical oversight and on HRWeb, a research information management platform. WAHO helped launch a regional network of health research institutions to improve collaboration between regional participating institutions. Further, WAHO developed strategic research partnerships and mobilised additional funding to support the program. The program supported 24 health research projects. High staff turnover, weak institutional capacities and ineffective collaboration were some of the challenges encountered during program activity implementation.

**Conclusion:**

The regional collaborative approach to health research development using this framework was effective given the challenges in the West African region. The achievements particularly with improved research partnerships and funding helped strengthen local health research environments. This highlights WAHO’s role and the common experiences in the West African region in improving health research.

## Background

In 1998, the heads of the 15 states of the Economic Community of West African States (ECOWAS) established a regional health institution, the West African Health Organization (WAHO), to improve health systems and address the common health challenges faced in the region through coordination, collaboration and cooperation among the member states [1]. The ECOWAS countries are Benin, Burkina Faso, Cape Verde, Côte d’Ivoire, The Gambia, Ghana, Guinea, Guinea Bissau, Liberia, Mali, Niger, Nigeria, Sierra Leone, Senegal and Togo. The ECOWAS Assembly of Health Ministers oversees the technical work of WAHO. The challenges that required multinational collaboration included epidemics, counterfeit medication trade and the harmonization of policies to address their common health problems. The development of health research in the region forms the basis for using evidence to bring about improvements in health systems and to address some of these challenges. Thus, research is of paramount importance for WAHO to achieve its mandate.

The National Health Research System (NHRS) has been defined by WHO as a system that provides the governance, the development of research capacities, knowledge generation and evidence utilization mechanisms as well as the accompanying sustainable financing mechanisms of any country’s health system for the conduct of its health research activities [2]. The NHRS is an important component of any modern health system, and defines the environment in which health research is nurtured and performed. NHRSs have been found to be weak in many regions across African [2]. WAHO’s initial strategic plan (2003–2007) did not take the enormity of this weakness into account and thus did not focus specifically on research, but rather on the diseases of greatest burden in the region [3]. However, a second WAHO strategic plan (2009–2013) [4] was developed in consultation with development partners and covered the following broader areas: WAHO’s institutional development, coordination and harmonization of policies, health information, promotion and dissemination of best practices, development of human resources for health, medicines and vaccines, development of traditional medicine, diversification of health financing mechanisms, and the development of research [4].

Several papers have previously described the varying degrees, and weaknesses, of African research capacity strengthening efforts [5–8]. This is especially so for the West African region compared to others in the eastern and southern regions of the continent. West Africa has been limited in terms of scientific publications [9–11], research capabilities [9, 12], research training capacities [13], and capacity for research resources mobilization [14], as well as the ability to develop research collaborations among countries in the region, particularly across linguistic barriers [9, 10, 13–15]. The environment within the Ministries of Health (MoHs) in the ECOWAS region has not been favourable for research development due to a lack of adequate qualified human resources, funding, and policy or strategy for research development [11]. Other regional organisations around the world, such as the Pan American Health Organization region [16], and WHO [17] have used different models that focus on capacity, priorities, standards and research translation to address the research challenges. Institutions such as the Special Programme for Research and Training in Tropical Diseases and the European & Developing Countries Clinical Trials Partnership have taken a more targeted approach at working with individual projects in low- and middle-income countries (LMICs) instead of supporting the whole NHRS system or using regional collaborative approaches for research. Many LMICs also do not have clearly identified NHRS priorities to guide their development.

Considering these challenges and the various frameworks available [18], WAHO, using the Pang et al. [11, 19] research capacity development framework, instituted a research development program for the West Africa region for the implementation of its second strategic plan (2009–2013) [4]. This framework was originally developed by Pang et al. [19] to help countries faced with similar challenges to focus on the following four key interrelated operational components – (1) stewardship, (2) financing, (3) creating and sustaining resources, and (4) producing and using research – and was original intended to be applied at country level. WAHO adapted the framework by adding a component on partnership development to guide the development of its regional research program. Partnership development is central to the operations of WAHO as a regional institution.

At the end of the period for the second strategic plan, WAHO needed to share the experiences and document the strengths, weakness and challenges during the implementation of this research development program for posterity. This would guide future work by WAHO or any organization intending to use the Pang et al. [19] framework.

## Methods

This is a retrospective review of WAHO’s research program, which was developed using the framework as a guide over the period of implementation by WAHO and its partners. Every activity conducted with WAHO’s involvement results in the generation of a technical report and, often, a financial report produced by WAHO staff and/or implementing partner. The data used for this paper was collated from a review of all the technical and financial reports of the research activities (approximately 150) and an independent report commissioned by WAHO in 2014 to evaluate the research program. This information was triangulated with field experience and exchanges between authors and stakeholders to summarise the implementation of the activities, results and the challenges encountered during the implementation of the research development program.

### The independent evaluation

To obtain an external viewpoint, an independent consultant was engaged to review all the research activities WAHO had implemented under the framework during this period (2009–2013) and another specific project funded by the International Development Research Centre (IDRC), which focused on the following under-developed countries, Guinea Bissau, Liberia, Sierra Leone and Mali, in 2014 [20]. Working with language translators, the consultant independently interacted with the Directorate General and staff of all the departments in WAHO and many of the key stakeholders at regional and country level. Over 180 in-person or telephone/skype interviews were conducted to collect the data using semi-structured interviews from June to August 2014. These included the directors of health research centres, persons in charge of research in the ministries of health, education, and science and technology, beneficiaries of WAHO funding for scholarships or projects, researchers, technical and financial partners, and the ECOWAS Commission staff. A team of two other independent consultants supported the interviewing process during this period. The consultant independently reviewed the technical and financial reports available for all the activities during the period. WAHO, along with its country and regional partners, validated the evaluation report in February 2015 during a regional meeting.

### Analysis

Analysis of the technical and financial reports focused on summarising and highlighting the trend in the strengths, weaknesses and challenges of the different activities of the five components of the framework during the period under review. The analysis had been presented as part of annual ECOWAS reports during the period and correlated well with the independent report and feedback given by the 80 representative partners during the February 2015 validation meeting. The framework approach, implementation, results and challenges of the framework used to improve health research are presented herein.

### Design of the research development program

The Knowledge for Better Health framework described by Pang et al. [19] is used to define the boundaries, components, goals and functions for health research systems for the advancement of scientific knowledge and ultimately the utilization of that knowledge to improve health and health equity. This framework has four functional components: stewardship, financing, creating and sustaining resources, and producing and using research. Table 1 summarises the scope of activities for WAHO’s research development program under the Pang et al. [19] framework adapted by WAHO [11].

**Table 1:** Modified Pang et al. framework [11]

Components of the framework	Component definition and implementation actions
Stewardship	*Define vision for national health research systems, research priorities and improve ethical standards* • Supporting countries to develop their national research policies, strategies and plans • Improving research ethics training and oversight
Financing	*Secure research funds and allocate them accountably* • Provide research projects financing on priority health needs • Provide resource mobilization training downstream to implementing partners
Creating and sustaining resources	*Build, strengthen and sustain human and physical capacity to conduct, absorb and utilise health research* • Support individual and institutional capacity development through scholarships and training programs
Producing and using research	*Produce, translate and communicate valid research to inform policy, strategies, practices and public opinion* • Promoting research dissemination (journal and conferences) • National, regional and global advocacy for using evidence-based policies and practices • Supporting the development of research on the health systems
Partnerships*	*Develop and improve partnerships to promote collaborative work to address common health issues* • Improve partnership with institutions, ministries, technical and financial partners • Support the development of research networks

*Partnership was not included in the original Pang et al. framework

The WAHO research development program had activities addressed to each component of the adapted framework.

To support countries in improving their stewardship functions (governance, management) of their NHRS, WAHO sought to provide technical support and targeted financing to the various member countries to develop their NHRS structures and guidance documents. WAHO gave priority to Liberia, Sierra Leone, Guinea Bissau and Mali, which had relatively underdeveloped NHRSs. Following a situational analysis at the beginning of the implementation, it became evident that these lacked important national governance structures and/or strategic documents (for example, policies, plans and/or priorities). A second area of activities programed under the stewardship component focused on strengthening ethics committees in member states through training and the promotion of HRWeb, an online health research information management platform. This platform would provide an overview of all the NHRSs in the region and make each country’s basic research laws, policies, plans, priorities, guidelines, and ethics review and research institutions information freely available online.

Through implementation of the financing component of the framework, WAHO provided additional financing towards various priority research projects. These involved calls for proposals, financing projects developed by participants on completion of health system research methodology training sessions, and special projects often requested by the various MoHs. Since health research projects are usually carried out at the local level, a regional resource mobilization training was also provided for participants from across the region. These provided participants with the opportunity to learn effective local fundraising mechanisms.

For the component on resource creation and sustaining, WAHO worked to develop individual level capacities (scholarship support program, support several training workshops) and institutional level capacities to create opportunities for collaboration, training and exchange between health research institutions across the region.

Under the production and utilization of research component, WAHO supported the creation of the West African Research for Health Journal and a bi-annual regional scientific congress on research results dissemination during this period in order to address the limited region-wide dissemination channels for the research results being produced. WAHO took up an active advocacy role in several global fora on the use of research to inform policy and practice. WAHO has actively supported the regional discussion on the development on various aspects of health systems research itself in the region through several regional meetings and supported the development of a regional project to promote health systems research development, teaching and utilization in West Africa.

For partnership development, WAHO worked with and through many of the key research stakeholders in the region and beyond, such as IDRC, Council on Health Research for Development (COHRED), the Wellcome Trust, and the European Development Clinical Trials Partnership, among others.

The implementation of all the activities as described under the framework was performed through an annual planning process by the WAHO Health Research Unit. WAHO committed three million US dollars of its budget to implement the activities under the framework during this period. WAHO worked with its partners during regional meetings, country visits, conducting situation analysis, training sessions, and providing financial and technical support to the research stakeholders in member countries, particularly through the MoHs, research institutions, ethics boards and other relevant partners engaging in research activities across the West African region. All the activities were directly aligned to WAHO’s vision and goals of establishment.

The triangulation of the information from the original technical and financial reports of the project activities, the independent report, and the authors’ experiences interacting with stakeholders is used here to provide a descriptive overview of these activities under the framework. The authors were directly involved in the adoption and implementation of this framework and their perspectives are reported as part of the results.

## Results

### Stewardship (defining a vision or priorities for NHRS and improving research ethics)

In 2009, WAHO conducted two regional health research situational analysis meetings with its implementing, technical and financial partners [21–23]. In September 2009, WAHO organised a meeting with the heads of the research centres from across the ECOWAS region to better understand the research structures in terms of their major areas of research, human resources, laboratories capacities, needs, partnership, funding sources and participating networks. This meeting affirmed that weak technical and managerial capacities of health research centres, the lack of sustainable funding, and weak intra-regional collaboration between research centres continues to plague the region. One of the main recommendations made to WAHO from that meeting was to support the creation of a regional network of research institutions to help facilitate collaboration between researchers in the region. The West African Health Research Network (WAHRNET) [24] was created, with 30 research institutions and 22 medical schools in 2010, and recognised by the ECOWAS Assembly of Health Ministers in 2012.

WAHO, with technical assistance from COHRED, conducted a second analysis of the NHRSs of all the countries in the region in December 2009 [23]. This involved the heads of research units of the ministries of health, education, science and technology, and the head of one major health research institution from each of the 15 member countries, who provided further clarification of the status of research in their respective countries. The meeting conclusions pointed to important deficiencies in coordination, governance and management structures, policy framework development, utilization of research results, capacity development, political support to research for health, and availability of financial resources. In summary, both meetings showed that many of the NHRSs were in a weak state lacking funding and strategic national direction [11, 22, 25]. This formed the basis for the development of a regional project to assist four of the weakest countries to begin rebuilding their systems (Mali and three post-conflict countries, Liberia, Sierra Leone, and Guinea Bissau) [11, 22, 25]. The objectives of the project were to strengthen the capabilities of the national health research systems and to promote the regional use of HRWeb, an online research information management platform in those countries. Other countries (Côte d’Ivoire, Niger, Togo, and Nigeria) also received technical and financial support from WAHO for the strengthening of their NHRSs based on the outcome of the initial situational analyses. The support WAHO provided also allowed for eight countries (Côte d’Ivoire, Guinea Bissau, Liberia, Mali, Niger, Nigeria, Togo and Sierra Leone) in the region and one research centre to develop and adopt various important strategic research guidance documents (policies, research development plans and national research priorities) to guide their work [26].

Research governance, namely the structures and systems for setting standards, promoting and using health research at the national level, is an important pillar in developing the NHRS. Therefore, in February 2011, WAHO had another review of the role of research within the MoHs of all the member countries, often the main oversight body and consumer of health research, on the specific needs required for promoting research and its use within the MoHs [11]. The results showed several weaknesses that, to varying degrees, included the absence of research guidance documents and training. Some MoHs had no budget lines dedicated to implementing or promoting research despite being signatories to the Bamako and Mexico declarations. There was the lack of consultation for research input into framing national health policies. Many national ethics committees’ members and research managers in the MoH were ill-equipped to carry out their duties and did not benefit from continued training [11]. To improve this situation, WAHO supported some research managers within the MoH to participate in a regional training program in the area of research management.

During the 5-year period, WAHO has worked with COHRED as a strategic technical partner [11, 21–23] to implement many of its activities. COHRED has provided its expertise to help several countries in the region to improve their overall health research environment by providing training in ethics, facilitating the countries to draft their research priorities and policies, etc. For example, COHRED provided refresher training for two ethics committees in Liberia to improve their capacities to conduct ethical review. This collaboration has produced many other benefits, including the setting up of an online research information-management platform for the region, HRWeb (waho.healthresearchweb.org) and training of over a 130 people across the region to use it.

### Improving research financing

To ensure adequate funding for the implementation of the program, WAHO, through its regional budgetary allocations, committed US$ 3 million seed money over a period of 5 years to support the development of research in all the member countries [27]. In 2011, the IDRC supported the development of the regional project with a sum of CAD$ 750,000 and, in 2010, the Wellcome Trust supported the financing of the first scientific congress of the regional WAHRNET with GBP 17,000. The joint Tropical Diseases Program of the UNICEF/World Bank/WHO consortium also supported WAHO’s research program in 2013 with approximately US$ 49,500 [27]. The Wellcome Trust and the IDRC also supported the second scientific conference of the WAHRNET with funds. WAHO used these funds to supplement its own funding to implement the strategy and provide regional activities that also foster collaboration within the region.

Over the 4-year period, WAHO provided over US$ 857,000 direct funding to 24 priority research projects in various arears of the health system [26]. These projects were in the areas of HIV disease and tuberculosis (n = 9), the quality of care (n = 2), health financing (n = 3), trachoma (n = 1), sickle cell disease (n = 1), malaria (n = 3), maternal health (n = 1), medicinal plants (n = 1), non-communicable diseases (n = 1), typhoid fever (n = 1) and dengue (n = 1).

Various mechanisms were used to select research projects for funding. This included financing projects developed by health system research methodology training participants (16 projects), financing projects in response to a call for proposals made by WAHO on operational research (five projects), and a few specific research projects requested by the MoH to improve the implementation of a specific country’s health program (three projects).

Many of the projects’ results have been reported locally at the country level, particularly to decision makers. Four articles from these projects have also been published in international peer-reviewed journals. For example, in Niger [26], the results of the funded research project were used to inform the launching of the national sickle cell disease program. In the Gambia, the research project showed that the financial motivation made by the Global Fund helped in the retention of health personnel in the public sector.

Supplementary technical and financial support from other partners has been important and made available to countries because of WAHO’s leadership, strategic program and financial management. It is often typical that countries undergoing political instability lose their donor funding support. During such difficult times, affected countries need the most support to prevent the collapse of their health systems, including their health research efforts. For example, during the period under review, when Mali had its political crisis in 2012–2013, many donors supporting research and development projects pulled out of that country [26]. WAHO is sometimes one of the key ways to continue to support the NHRS of a member state to prevent it from total collapse.

Some difficulties identified during the period generally relate to the financial management processes for the projects, bureaucratic delays and the lack of effective project management planning. For example, funds sometimes go through public general services accounts before arriving at the researcher’s institutions, causing delays to accessing the funds for implementation. Banking errors sometimes occur and delay the project by up to 6 months at the country level. At the institutional implementation level, disagreements between investigators, low or lack of capacity of research teams and the unavailability of key investigators were recorded to have led to challenges. Some investigators were juggling too many other roles in their institutions, leading to delays in the implementation of projects because of their limited availability to either carry out or supervise their projects. Since 2009, WAHO sometimes provides financial and technical management oversight of some other donor funded projects. This added oversight function has enabled the projects to pass frequent routine external donor audits. WAHO does not have permanent in-country offices in the member countries and therefore several measures are instituted to ensure responsible use and accountability of the funding. Some of these include allowing performance contracts for the projects to be signed with the MoH or country supervision to be performed by WAHO monitoring/evaluation and financial teams during their routine country visits. These approaches help resolve some of the difficulties on the ground and ensure researchers/institutions pay more attention in managing WAHO/donor funds.

### Creating and sustaining resources

WAHO has used several approaches to support the human resource capacity building for health research in the region [26, 27]. At the individual researchers’ level, different approaches were used, including (1) the provision of scholarships for research development training for health researchers in the countries, (2) supporting the Medical Research Council of the Gambia conduct distance-training programs, and (3) supporting some countries’ (Togo, Burkina Faso) requests for scholarships to meet their specific research priority needs (epidemiology certification training for staff in the university). Many of the individual requests are channelled through the MoH to WAHO. During the period of implementation, 27 scholarships were granted through this process. These scholarships permitted recipients to undertake graduate and postgraduate training in public health (two PhD), epidemiology (nine MSc), health economics (one MSc), health demography (one MSc), biology (seven BSc/MSc), health information and informatics (two MSc), and certificate courses in laboratory studies and good clinical practice (five).

The second approach used by WAHO consisted of the organization of training workshops. Fifteen workshops were co-organised or sponsored by WAHO over the period (2009–2013) comprising of training in health systems research methodology (n = 5), training in resource mobilization performed in partnership with the WHO Regional Office for Africa (n = 3), on ethical oversight (n = 1), and on the use of the research information management platform, HRWeb (n = 6). Course participants were mainly drawn from the MoH, universities or research institutions. About 43 teams across the region and 14 WAHO staff have received training through workshops in resource mobilization as have 88 leaders from the MoH and the research institutions from all the West African countries on research methodology for health systems research. Over 130 researchers, research programs managers and information technicians also received training on the use of the HRWeb platform for managing health research information.

For example, to assess the impact of these capacity development activities, post-training feedback obtained from the participants who trained in the resources mobilization session indicated that they have had improved engagement in resource mobilization efforts for their respective institutions. That training also enabled WAHO to improve its own capacity to mobilise and manage additional funds to improve its ability to implement its programs.

There were a number of challenges for scholarship recipients. The WAHO scholarship grants initially covered only academic and residential costs for the students for a year, while some training programs were for a 2-year period. This was resolved by extending it to 2-year bursaries for such programs. Another major challenge was the reappointment or re-entry of trainees into their countries after their training, as WAHO’s scholarships are not conditioned upon guaranteed employment on completion. Indeed, some fellows had no secure work appointments after their studies despite the reported shortages of the human resources capacity in the region, therefore guaranteeing reappointment from candidate’s employers should be a precondition for future award selection.

### Promoting the dissemination and utilization of research results

Attention was given to activities that would be of significant impact under this component of the framework. WAHO focused on supporting the dissemination of research results within the region to avoid study duplication, promote policy uptake and translate policy into practice while efficiently leveraging the limited resources across the region. WAHO therefore supported the organization of two regional scientific congresses for the WAHRNET and three other national level scientific congresses. WAHO also supported WAHRNET to launch the *West African Journal of Research for Health*, a peer-reviewed multi-lingual journal to promote the work of regional researchers. With that same aim, WAHO also helped fund the publication of other regional research reports, including a document on the composition of local foods in West Africa [26].

### Partnerships development

WAHO has traditionally developed many strategic partnerships with the MoHs, health institutions and with its technical and financial partners to help achieve its objectives, particularly because of its limited in-country presence, limited funding and small number of staff to cover the 15 countries and 300 million people. The in-country partners who carry out the research activities and are able to assess their local research environments, provide the essential feedback through their interaction and reporting to WAHO, which operates at the supra-national level. These partnerships allow the countries to share information at regional level on their activities, resources and challenges aimed at collectively improving the research environment within the region through collaboration. For example, specifically, these partnerships have allowed decision-makers and researchers to appreciate their challenges and those of their neighbours. It has also allowed decision makers and the researchers to network with their counterparts working on similar issues within the region. WAHO has been able to represent the collective interests of the region in many international research settings where participation by all the countries was not possible for reasons such as cost or linguistic barriers, and include Health Systems Global Symposium and the African Advisory Committee on Health Research and Development, amongst others.

In May 2010, WAHO organised a regional forum for some of its health research partners in Dakar with the support of COHRED. This provided the first opportunity for a wide representation to review the region’s NHRS since the set-up of WAHO. The meeting helped to identify the priority areas of action and some of the prevalent common donor concerns hindering partnership in the region, including communication challenges, financing, information sharing, etc. WAHO also shared its vision with partners and this resulted in several partnership projects between agencies like the Wellcome Trust and the IDRC who provided financial and technical assistance to countries aimed at developing their research systems.

### Other general challenges

Many country-level participants at meetings often solicited WAHO to support and advocate on their behalf with the leadership in their countries in order to improve, with practical steps, their commitment to improving their research environment. Although health ministers, on behalf of their countries, had promised to allocate 2% of their health research budget and 5% of the budget of the program and projects to research, in some countries, there is no budgetary appropriation for research within the MoHs and there is the lack of collaboration between researchers, program managers and policymakers [11]. Some countries contract out their research programs or project activities to consultants instead of using their own research institutions and universities to pursue such inquiry. Contracting with their own national institutions supports the building of local capacity in these institutions and the additional funding that goes along with it [11, 28]. Further, some research institutions do not have sufficient capacity to implement research projects. Our experience with the funding of small research projects showed technical and managerial weakness in some of the institutions due to lack of research managerial expertise.

Ethical oversight is also another undeveloped area in some of the countries. Where ethical committees exists, there is little or no training provided for members to perform their roles effectively. Many committees are under-resourced and few deploy modern research information management tools for their efficient functioning and ease of interaction with researchers [29, 30].

Several research facilities also have difficulties with internet access and library services. The WHO Health Inter Network Access to Research Initiative (HINARI) has provided free online access to many full text journals to member states who qualify to participate as LMICs. Research takes place in the many communities in the region where there are challenges with constant electricity and water supplies, communication, transportation and housing needs. These tend to affect all aspects of the studies carried out and thus the research environment. Many of the countries are among those that consistently rank high on the global lists of perception of corruption and have extensive bureaucracies that further exacerbate their challenges. Manual administrative processes with poor archiving and data retrieval systems are the norm in many countries; this is typical of hospital records, ethics review processes and management of financial records in many NHRSs. Travel and physical entry into any one of the ECOWAS countries also require unique processes, understanding of the culture and ways of conducting business. Such knowledge is critical for international partnership in global clinical trials, for instance. It is also sometimes difficult to share and understand the unspoken intentions among collaborating partners implementing a research project because of local nuances. For instance, the potential financial incentives of a project may cause a project team to over-estimate its capacity and availability of key personnel. Another significant outcome of reviewing the implementation of this program has been the development of a new West African Regional Strategic Plan for Health Research, which was adopted by the ECOWAS Assembly of Health Ministers at their 17th Ordinary Meeting on 8 April 2016 in Bissau, Guinea Bissau.

Another challenge was achieving full participation of the country representatives and other key stakeholders during the WAHO regional meetings, particularly during the development of the common vision and priorities. This may be attributed to factors such as travelling difficulties within the region and linguistic barriers. There are also frequent changes of research managers and policymakers, particularly in the MoHs across the region, hindering continuity in work progress and participation in regional meetings. Post-meeting dissemination and consensus appropriation at the country level is weak, limiting ownership and stewardship of the NHRS at the country level.

Overall, the West African countries continually and collectively through their MoHs and representative health research institutions, committed to the work of WAHO by their continued engagement with WAHO and endorsing its strategic plan [27].

## Discussion

WAHO has been able to support the NHRSs and thus improve the health research environment in West Africa during the 5-year period under review using the adapted Pang et al. [19] conceptual framework as a guide to implement its activities. These results include the development of health research policies, plans or priorities in eight ECOWAS countries, the funding of 24 research projects, providing for 27 human resources training scholarships, the creation of a regional network for health research institutions, a regional scientific journal, and initiating scientific congresses for results dissemination of the regional level, to name a few. The impact of these activities have also improved the dialogue between the different health research stakeholders at the country and regional levels. These also became a catalyst for other national level developments in NHRSs in the region [26]. The results of the WAHO program has helped to begin to address some of the main weaknesses in the West Africa region identified by earlier authors relating in the ability to conduct quality research, resource mobilization, and having national research policy documents [2, 9–13, 31, 32]. The activities addressed the gaps identified during the situational analysis conducted at the beginning of the implementing period. Obviously, several political and armed situations that occurred in the region during the implementation period took its toll on the ability to improve the research environment in those countries. This, sometimes coupled with frequent changes in institutional and national leadership, affected program implementation. These security issues, for example, hampered field visits for the WAHO team and its partners, meetings for researchers in general and heightened donor security concerns.

It would be unrealistic at this stage to want to realise all the benefits and impact of these regional programs on the health status of the peoples and health systems of the region since building the NHRS is a long-term process. During this period, several opportunities were identified to create transnational projects that work across the linguistic divides in the interest of the region. Some differences amongst the countries in the region, such as level of development, particularly of the health system, sometimes affect the ability to collaborate on projects to improve the health research environment. These differences are reflected in such indices as the human development index and corruption perceptions index [33–36]. Access to and the availability of research funding across the region is highly variable. For example, countries like Ghana, Nigeria and Senegal are able to access more global funding for their research activities because of their record of accomplishment of developing and sustaining partnerships. Burkina Faso’s establishment of a national fund for the advancement of research is an example of an encouraging development in the region. The access to funding creates and explains some of the variability in the development of the NHRS amongst the countries in the region and hence the research environment.

Even though English is broadly acknowledged as the language of global scientific research, that also creates a barrier for accessing and utilizing research results, training opportunities, and international conferences for a broad swath of researchers and decision makers who are unable to communicate in English. Only five of the 15 member countries in the region are Anglophone. This linguistic barrier is especially so for local traditional medical practitioners and researchers. Further, the 2015 estimated total population literacy rate in most of the countries is less than 60%, particularly in the francophone countries [37]. The historical linguistic divides in the region amongst the three predominant languages of English, French and Portuguese and the inability of many researchers to communicate comfortably in the other languages have, to some extent, limited meaningful regional research collaborations to address the common issues affecting their health systems. This has also hindered personnel from research training opportunities available in the region by limiting access. For example, if a francophone research training program became available in the Cote d’Ivoire, participants from neighbouring Liberia or Ghana would not be able to access it and would have to travel to the United Kingdom or the United States of America for such training. WAHO has had two language-based programs to help address some of these difficulties; these are the Young Professionals Internship Training Program and the Fellowship Program for Professional and Linguistic Exchange for health professionals [1, 27]. These two language immersion programs help health professionals learn other languages and facilitate collaboration between health professionals by placing them in health/research institutions in another country for a period. The outcomes are more collaboration between researchers and improving technical and communication skills for researchers.

The health research environment is not separate from the wider society. Therefore, the effects of the economies, politics, security and disasters have an impact on health research activities. Some institutions are in such a delicate state that they do not have the capacity to withstand any major shocks to their operations. For example, Mali, which had several robust research institutions in the country, came under immense stress following internal conflicts during 2012. Donor funding in such circumstances is immediately curtailed, further worsening their precarious situations.

The human resources capacity for the development of NHRSs in the region is inadequate in terms of quality, distribution and quantity of staff in some places. The lack of successful career paths for young researchers, the limited training and lack of mentoring opportunities, the inadequate remuneration and the lack of funding for research activities have been found to de-motivate researchers [11]. The researcher thus spends more of their time on other income-generating activities such as consultancies, clinical, administrative and teaching duties to supplement their income. For the researchers and their institutions, the infrastructure required to carry out their activities is often inadequate. There is a very small bio-medical research engineering industry in the region to support the development and maintenance of the research infrastructure and therefore equipment often needs to be imported and serviced from developed countries. On the broader governance issues, some of the countries in the region have or are in the process of developing broader national research policies, which would help guide the national planning, prioritization, and financing of their research activities.

The challenges of the implementation of the funded projects in the countries [32, 38, 39] have brought to the fore the weak administrative and technical capacities of some of the research institutions. These weaknesses call for helping the research institutions to improve through research management training, recruitment, training for the adequate human resources [39] and improvement of the working conditions of researchers [11].

Working with all the research stakeholders at the country level and collaboration with the other technical and financial partners contributed to the success of the WAHO research program. This approach instilled confidence in stakeholders and brought them the necessary support to facilitate their own work [5]. The many different meetings allowed researchers and research managers to identify their common needs and to collaborate and propose solutions to address these needs. The different fora created to pursue these collaborations also strengthened dialogue between researchers and decision-makers and spurred the establishment of WAHRNET [24], aimed at improving researcher interactions and the training of researchers. The various fora helped to reduce the language barriers between researchers, thus allowing for more regional level collaboration.

Several ECOWAS member countries have been able to complete the development of their research policy documents, research priorities and implementation plans, which would serve as a road map for the countries and partners to assist in their implementation for their NHRS development. As many countries now have their own policy and priority documents, WAHO has now developed a harmonised regional strategy to provide direction and vision for health research development in the whole region [20]. This new WAHO regional research strategic plan document (2016–2020) is in the process of being adopted by the ECOWAS Assembly of Health Ministers on behalf of the Heads of States. These national and regional strategic plan documents are important tools in the mobilization of funds for research and in negotiations with partners. WAHO would continue to work to support countries to implement their plans. Areas such as the strengthening of ethics oversight, coordination and developing a system of monitoring and evaluation using agreed indicators for the performance of countries and the region as a whole were to be taken into account in the new WAHO research strategic plan [20]. The political will and understanding of the leadership of the ECOWAS institutions helped to achieve this success. The ECOWAS budgetary support for implementing the regional research program during the period was helpful. Many of the activities accomplished with the funding could not have been possible with the solitary efforts of any one country. Through this funding stream, WAHO was even identified as a potential funder by some researchers or some international funders in the research field in Africa [38] and increasingly sought to share its experience of being a funder from and working in a resource-poor region. Improvement in the management and growth of this funding has become very important to help cope with the many identified needs that can best be addressed at the regional level and to ensure sustainability. The experiences of performing this role led to a unique learning exercise allowing WAHO to develop its ability to make calls for research proposals, adopt a review process, provide grants and be more sensitive of gender inequality of research capacity in the region as an institution.

Several approaches can be used to build capacity in the region that is evident in initiatives such as the strengthening academic training and towards short courses to train stakeholders in the field [13, 32, 39–43]. WAHO’s vision is also the ability to develop more transnational training programs and collaborative research activities that focus on addressing the regional health research priorities and health needs.

There is some elevation of political commitment towards the development of health research and the provision of practical platforms for collaboration, research funding, training of research professionals and the translation and utilization of research results in health programs [27, 41]. This same commitment is also seen in mainstream traditional medicine research practice in the West African region [1, 9, 18, 26, 27]. Improvement is also required in the area of traditional medicine and pharmaceutical research and services to improve indigenous solutions to healthcare delivery [28, 30, 31, 39, 42].

## Conclusion

This West African experience of using a regional approach to support the development of health research across this region was effective. Overall, the achievements of the WAHO program, especially with the mobilization of partnerships and the financial and technical support given to countries for the strengthening of their own NHRS and thus their local health research environments, shows how regional level collaboration amongst countries with adequate funding can work [41, 42]. Despite the difficulties in the environment, health research can develop in Africa by focusing on the gaps identified in situational analysis in the countries and, where possible, on the region as a whole [39]. WAHO’s role and experiences in promoting stakeholder collaboration and mutual learning at the country and regional level is important in improving health research in the region.

This paper contributes to the body of experiences of how to adapt a guidance framework to design and implement a research development program. The Knowledge for Better Health Development Framework proposed by Pang et al. [19, 44], mainly for LMICs, was used here to guide the development of a research program at the regional level to improve the environment in which research is conducted. To date, there is insufficient evidence published to attest that this has ever been done; therefore, further work is needed on the effectiveness of the use of development frameworks, particularly in LMICs.

### Ethical approval

No ethical approval was required for this work.

## Abbreviations

COHRED: Council on Health Research for Development
ECOWAS: Economic Community of West African States
IDRC: International Development Research Centre
LMICs: Low- and middle-income countries
MoH: Ministry of Health
NHRS: National Health Research System
WAHO: West African Health Organization
WAHRNET: West African Health Research Network
